# Two Haplotypes of *Aedes aegypti* Detected by ND4 Mitochondrial Marker in Three Regions of Ecuador

**DOI:** 10.3390/insects12030200

**Published:** 2021-02-27

**Authors:** Patricio Ponce, Sofía Muñoz-Tobar, Andrés Carrazco-Montalvo, Stephany D. Villota, Josefina Coloma, Chunling Wang, Susan Holechek, Varsovia Cevallos

**Affiliations:** 1 Instituto Nacional de Investigación en Salud Pública, Gestión de Investigación, Desarrollo e Innovación, Quito 170136, Ecuador; wpponcey@yahoo.com (P.P.); munoztobarsofia@gmail.com (S.M.-T.); andres.carrazco@hotmail.com (A.C.-M.); stephany.villota.v@gmail.com (S.D.V.); 2 Simon A. Levin Mathematical, Computational and Modeling Sciences Center, Arizona State University, Tempe, AZ 85281, USA; Susan.Holechek@asu.edu; 3 Division of Infectious Diseases and Vaccinology, School of Public Health, University of California Berkeley, Berkeley, CA 94720, USA; colomaj@berkeley.edu (J.C.); ocean0818@gmail.com (C.W.); 4 School of Life Sciences, Arizona State University, Tempe, AZ 85281, USA

**Keywords:** *Aedes aegypti*, haplotype, Ecuador, ND4

## Abstract

**Simple Summary:**

The yellow fever mosquito, *Aedes aegypti*, is a widespread species associated with the transmission of vector-borne diseases across tropical and subtropical areas of the world. The genetic variability of its populations has been assessed with the use of several molecular markers to understand aspects of the population dynamics and their implication in disease transmission. However, the genetic diversity of Ecuadorian populations of the vector have not been investigated. In this study, we evaluated the genetic diversity of Ecuadorian populations of *Ae. aegypti* from 17 sites (Galapagos Islands, Amazon basin, and Coastal regions). These analyses revealed the presence of only two haplotypes among the Ecuadorian population of the vector. Haplotype 1, appears to be related to previously reported haplotypes from America, Asia, and West Africa. While haplotype 2 is only related to samples from America. The genetic diversity of Ecuadorian populations seems to be low, according to different statistical analyses, which show only one main population across sampled localities and no effect of the main geographical barriers. Understanding the genetic diversity of local populations is a key element in vector control strategies.

**Abstract:**

*Aedes aegypti*, also known as the yellow fever mosquito, is the main vector of several arboviruses. In Ecuador, dengue and chikungunya are the most prevalent mosquito-borne diseases. Hence, there is a need to understand the population dynamics and genetic structure of the vector in tropical areas for a better approach towards effective vector control programs. This study aimed to assess the genetic diversity of *Ae. aegypti*, through the analyses of the mitochondrial gene ND4, using a combination of phylogenetic and population genetic structure from 17 sites in Ecuador. Results showed two haplotypes in the Ecuadorian populations of *Ae. aegypti*. Haplotype 1 was closely related to *Ae. aegypti* reported from America, Asia, and West Africa. Haplotype 2 was only related to samples from America. The sampled vectors from the diverse localities showed low nucleotide diversity (π = 0–0.01685) and genetic differentiation (FST = 0.152). AMOVA analyses indicated that most of the variation (85–91%) occurred within populations, suggesting that geographical barriers have little effect on the genetic structure of Ecuadorian populations of *Ae. aegypti*. These results agree with the one main population (K = 1) detected by Structure. Vector genetic identity may be a key factor in the planning of vector control strategies.

## 1. Introduction

Mosquito-borne diseases pose a significant risk to human populations, health systems, and the economy; with a higher impact in poor tropical countries [1]. The yellow fever mosquito *Aedes aegypti* (Linnaeus, 1762) is present throughout tropical and subtropical regions of the world [2], acting as the main vector of several arboviruses, including yellow fever (YF), dengue (DENV), chikungunya (CHIKV) and Zika (ZIKV)—viruses [3].

New world populations of *Aedes aegypti* originated in West Africa and spread to the American continent in the 16th century, during the European exploration and colonization period [3,4]. The epidemiological history of *Ae. aegypti* in the Americas started around 1600, with the introduction and the spread of a dengue-like disease [5], which resulted in the implementation of a regional plan for the eradication of the vector with the use of DDT between 1947–1970s. These programs failed to control *Aedes* populations, with massive re-infestations and several dengue outbreaks reported between 1971 and 2010 [5].

*Aedes aegypti* is the known vector of DENV, CHIKV, ZIKV, YF, and the Mayaro virus (MAYV). In Ecuador, dengue is the most prevalent mosquito-borne disease, with 79,599 cases of dengue reported in the past six years, followed by 35,685 cases of chikungunya [6]. The abundance of *Ae. aegypti* in Ecuador is associated with poor housing conditions and the lack of access to public services, such as piped water, sewage, and garbage collection [5,7]. Chemical control of the adults and larval instars in water sources is the main method of controlling populations of *Ae. aegypti* [8]. Some populations of the vector have shown medium to high resistance to insecticides used in vectorial control [9]. This insecticide resistance generates important implications in the control of populations of the vector and prevention of circulating arboviruses.

New World populations of *Ae. aegypti* seem to have a subset of the genetic diversity found in African populations, which display higher genetic diversity [4]. The genetic diversity of some South American populations have been assessed using a wide range of genetic markers (i.e., allozymes, nuclear, and mtDNA genes) [10,11]. Still, mitochondrial DNA (mtDNA) genes are widely used for the identification of genetic variants, dispersal patterns, phylogeny, and population dynamic studies of *Ae. aegypti* [12,13]. Despite the importance of *Ae. aegypti* as the main vector of relevant arboviruses, there is limited information regarding its genetic diversity, levels of genetic connectivity, and structure of Ecuadorian populations.

This study aimed to assess the genetic diversity of *Ae. aegypti* from 17 geographical sites located in Ecuadorian the Pacific coast, Amazon basin, and the Galapagos Islands, through the analyses of the mitochondrial gene ND4, using a combination of phylogenetic and population genetic analyses. With these analyses, we aimed to determine the number of haplotypes present, establish the phylogenetic relationship among local haplotypes, as well as with haplotypes from around the world. Lastly, we wanted to determine the levels of genetic connectivity and population structure by performing population genetic analyses with a subset of localities.

## 2. Materials and Methods

### 2.1. Sample Collection

Individuals of *Ae. aegypti* were collected between 2012 and 2018 in 17 sites (Table 1; Supplementary Table S1) during the rainy and dry seasons, as part of entomological surveys done for the SAVTEC project. Sites were visited at least twice during this period. Locality selection was based on the number of arboviral clinical cases reported by sanitary authorities [14], and regional representation (Pacific coast, Amazon basin, and the Galapagos Islands). The adult mosquito samples were collected by aspiration using the Procopack aspirator (John W. Hook, Gainesville, FL, USA). Adult individuals were preserved in 70% EtOH, while larvae found in artificial breeding containers, were transported to the laboratory for the adults to emerge. Samples were identified following Rueda (2004) pictorial keys for the identification of mosquitoes.

Outgroup taxa selection was based on the availability of sequences deposited at GenBank. For the analyses based only on Ecuadorian individuals of *Ae. aegypti*, we used *Culex quinquefasciatus* Say, 1863 as an outgroup (Supplementary Table S2). While for comparative analyses among ND4 reported haplotypes we selected 46 sequences of *Ae. aegypti* from: Colombia, state of Sucre [15], state of Antioquia, La Guajira and Meta [12]; Peru [16]; Bolivia [17]; Venezuela [18]; Brazil, states of Alagoas, Ceará, Mato Grosso do Sul, Paraná, Rondônia, and Sao Paulo [10]; state of Paraná [19]; and Brazilian Amazon [20]; Mexico [21]; Cape Verde, Santiago Island [22], Santiago, Fogo, and Brava Islands [23]; America, Africa, and Asia [13] (Supplementary Table S3). Voucher samples are deposited in the “Colección Nacional de Referencia de Vectores” at the National Institute of Public Health Research—Dr. Leopoldo Izquieta Perez (INSPI-Quito).

### 2.2. DNA Extraction, Amplification, and Sequencing of the ND4 Gene

Genomic DNA was extracted from 137 individuals using DNeasy Blood & Tissue Kit^®^ (Qiagen, Germantown, MD, USA), following the manufacturer’s instructions. A fragment of the mitochondrial ND4 gene was amplified using the primers ND4+ (5’-GTDYATTTATGATTRCCTAA-3’) and ND4- (5’-CTTCGDCTTCCWADWCGTTC-3’), following the amplification protocol as described in Gorrochotegui-Escalante et al. (2000) [24]. PCR products were detected by 1% agarose gel electrophoresis in TAE buffer, stained using SYBR^®^ Safe 10,000×. PCR products were sequenced using the Sanger sequencing method at three locations: Macrogen, Seoul, South Korea; UC Berkeley Sequencing Facility; and Biodesign Institute, CLAS Genomics Core at Arizona State University. Sequences were edited using Geneious Prime 2019.1.1 [25] and aligned using MAFFT v.7 [26]. Models of sequence evolution were obtained through jModelTest-2.1.10. [27], for each data set, where GTR + I + G model was in the 100% confidence interval for our data sets.

### 2.3. Phylogenetic Analyses

Phylogenetic analyses were used to determine relationships among individuals of Ecuadorian populations, as well as to determine if haplotypes present in our local populations match published ND4 haplotypes from across the globe (45 sequences, Supplementary Table S2). The construction of the phylogenetic trees was done using MrBayes on XSEDE [28] and RAxML-HPC BlackBox [29] through the CIPRES Science Gateway v.3.3 (phylo.org). Consensus trees were generated in PAUP4 [30], using a 50% majority rule. For Ecuadorian samples, a haplotype network was constructed in PopART [31], haplotype designation was confirmed in DnaSP [32]. Lastly, the percentage of identity of our local haplotypes was compared with reported ND4 haplotypes deposited in NCBI (288 sequences, Supplementary Table S3), using BLASTn at the National Center for Biotechnology Information (NCBI) (http://blast.ncbi.nlm.nih.gov/Blast.cgi; accessed on 26 October 2020).

### 2.4. Genetic Diversity and Population Structure of Seven Populations Ae. aegypti

The genetic diversity of *Ae. aegypti* was assessed using DnaSP [32] and Arlequin 3.5.2.2 [33] applying default settings. Parameters estimated included: haplotype diversity (h), nucleotide diversity (π), number of polymorphic sites (S), and number of migrants per generation (Nm). Tajima’s D (D) and Fu’s FS (F) neutrality statistic tests were calculated in DnaSP.

Seven populations of *Ae. aegypti* were selected for preliminary population genetic analyses. Locality selection was based on the number of individuals per site (>8 individuals) and region. Chosen sites represent the Pacific coast (Guayaquil, Lita, Machala, Cumandá and Borbón), Amazon basin (Nueva Loja) and Galapagos Islands (Santa Cruz). The level of genetic connectivity among these seven populations was determined by calculating FST values using Arlequin. Additionally, the number of migrants per generation (Nm) among populations and the analysis of the molecular variance (AMOVA) were calculated. The latest was used to test the effect of geographical regions (Pacific coast, Amazon basin, and Galapagos Islands) and the effect of major geographical barriers (e.g., Andean mountains) as seen for Ecuadorian vertebrates and invertebrate species [34,35,36]. The correlation between genetic (FST) and geographical distances were tested by a Mantel test performed in Arlequin using 1000 randomizations. Lastly, the number of populations (K) was tested for seven possible populations by using no admixture and 10 iterations per run in Structure 2.3.4 [37].

## 3. Results

### 3.1. Phylogenetic Analyses

**Figure 1:**
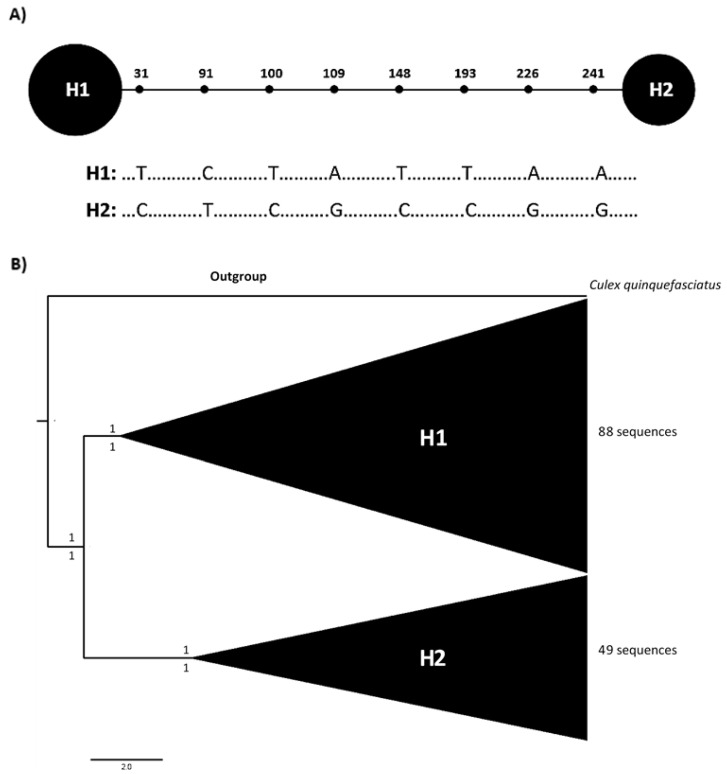
Genetic diversity of Ecuadorian populations of *Aedes aegypti* using ND4 mitochondrial marker. (**A**) Haplotype network for the ND4 gene for Ecuadorian samples of *Aedes aegypti*. Numbers represent mutational positions in a 275 bp fragment. Polymorphic sites with the specific transition. (**B**) Bayesian phylogenetic tree among 137 sequences of *Ae. aegypti* populations showing two haplotypes based on a 275 bp fragment of ND4 mitochondrial marker, using *Culex quinquefasciatus* as outgroup.

**Figure 2:**
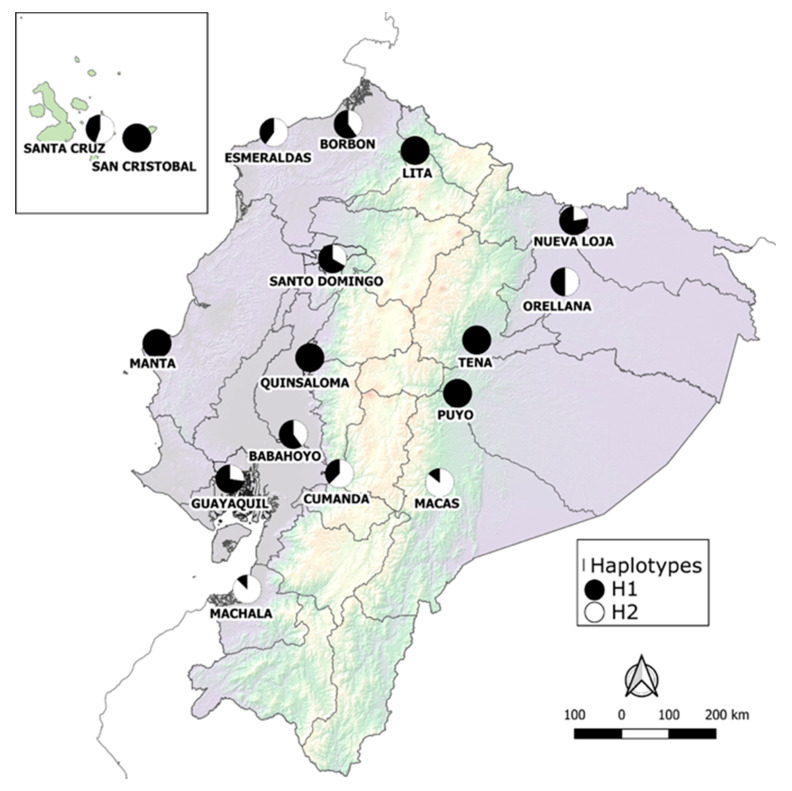
Map of distribution of the two haplotypes of *Aedes aegypti* populations in 17 localities of Ecuador. Pie charts represent the percentage of haplotype 1 (black) and haplotype 2 (white) present in each locality.

The portion of the ND4 gene analyzed was 275 bp long, with 8 parsimony informative sites, and two haplotypes detected in Ecuadorian populations of *Ae. aegypti* (Figure 1A, GenBank accessions MK905895—MK906025 and MW316314—MW316322). Phylogenetic inferences, generated through Maximum Likelihood and Bayesian methods using only Ecuadorian sequences, showed two well-supported clades (Figure 1B). Haplotype 1 was documented throughout sampled sites and was the most common haplotype recorded among Ecuadorian samples (64%). Haplotype 2 was only recorded in 11 out of the 17 sampled localities (Figure 2, Supplementary Table S1). When haplotype composition was compared among sites, six localities showed the presence of only haplotype 1, while the remaining 11 localities registered both haplotypes (Figure 2).

**Figure 3:**
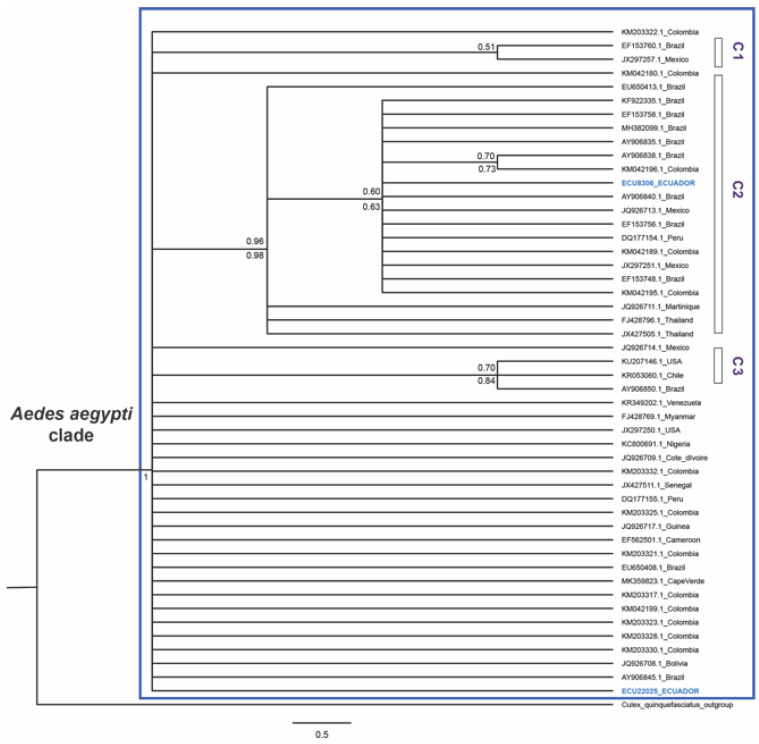
Maximum likelihood tree for ND4 mitochondrial marker of *Aedes aegypti* with haplotypes from around the world, including the two Ecuadorian haplotypes. Bootstrap support values for the ML tree are presented below branches and posterior probabilities are presented above the branches. Ecuadorian haplotypes are highlighted in blue. C1 refers to genetic cluster 1; C2 genetic cluster 2 and C3 genetic cluster 3.

The second set of analyses were performed to understand the phylogenetic relationships of the two Ecuadorian haplotypes, in reference to haplotypes obtained in previous studies. The resulting tree was a monophyletic clade for *Ae. aegypti*, supported by bootstrap values (Figure 3). However, the resolution of this tree was poor. Only three genetic clusters were recovered with the support of posterior probability and bootstrap values. The first cluster contained samples from Brazil and Mexico. A second cluster included the Ecuadorian haplotype 1, which contained samples from New World America (USA, Mexico, Colombia, Peru, Brazil, Bolivia, and Chile), Asia (Myanmar), and West Africa (Senegal, Guinea, Ivory Coast, Nigeria, and Cameroon) (Figure 3). The third cluster, included three samples from Chile, the US, and Brazil. Lastly, the Ecuadorian Haplotype 2 appeared to be located at the base of the *Ae. aegypti* clade with sequences from America (Mexico, Colombia, Peru, and Brazil), these only support the placement of these haplotypes within the *Ae. aegyti* clade (Figure 3).

The comparison of the two haplotypes with haplotypes of *Ae. aegypti* reported in NCBI was done using BLASTn. Haplotype 1 was identical to H1 in Brazilian Amazon [20], H11 in a broad study along Brazil [10], H13 in Mexico [21], H2 in Peru [16], H2 in the state of Sucre—Colombia [15], and H81 in states of Antioquia, La Guajira, and Meta—Colombia [12]; H4 in Bolivia [17]; H15 in Africa [13], and H6 in Santiago, Fogo, and Brava Islands—Cape Verde [23] (Table 2). No similarities were found with haplotypes reported in Paraná, Brazil [19], Venezuela [18], or Santiago Island—Cape Verde [22]. Haplotype 2 was identical to H10 in Brazilian Amazon [20], H2 in Brazil [10], H20 in Mexico [21], H1 in Peru [16], and H5 in America [13] (Table 2). No similarities were found with haplotypes reported in Colombia [12,15], Bolivia [17], Venezuela [18], Paraná—Brazil [19], or Cape Verde Islands [22,23].

### 3.2. Genetic Diversity and Population Structure of Seven Populations of Aedes aegypti

The overall nucleotide diversity of the Ecuadorian populations of *Ae. aegypti* was low (π = 0–0.01685; Table 3). This was supported by the overall value of the FST´s based on 7 populations, which suggested that there was low genetic differentiation among the analyzed populations (FST = 0.152). However, when these values were compared on a population by population basis, high levels of genetic differentiation were recorded among Lita and Cumandá (FST = 0.56), and between Machala and Lita (FST = 0.78; Table 4). In both cases, these localities are situated within the same region but separated by distance. The highest number of migrants per generation was found between Borbón (Pacific coast) and Nueva Loja (Amazon basin; Nm = 23.74), two sites with proximity to Colombia but separated by the Andean mountains. Followed by two port cities, Guayaquil (Pacific coast) and Santa Cruz (Galapagos Islands; Nm = 4.93), and by two localities within the province of Guayas, Guayaquil, and Cumandá (Nm =4.63, Table 4). For most of our data, FST can directly be related to migration [38]. Still, several sites show low FST values and a small number of migrants per generation. For example, Santa Cruz and Borbón, or Cumandá and Santa Cruz (Table 4). This pattern could be explained by the presence of similar allele frequencies within each population [39], and geographical distance between sites. Yet, the Mantel test performed to analyze the correlation between genetic (FST) and geographical distances, showed no correlation (*p* = 0.66, R2 = 0.00). Even though several variations of the AMOVA were used to test the effect of regionalism and geographical barriers, most of the variation was seen within groups (Table 5). Analyses performed in Structure to determine the number of populations showed one main population (K = 1), among sampled sites.

## 4. Discussion

This is the first study to analyze the haplotypic diversity of populations of *Ae. aegypti* from different regions of Ecuador. Our analyses showed that Ecuadorian populations of the vector have low haplotypic diversity, based on the analysis of the mitochondrial gene ND4. Only two haplotypes were identified among the analyzed material (Figure 1), where haplotype 1 is the most widespread haplotype, present in all sampled localities (Figure 2). In contrast, haplotype 2 was detected in 11 out of the 17 sites. Overall, there is no clear pattern of distribution for each haplotype, since both alleles are recorded in the Pacific coast, Amazon basin, and Galapagos Islands; where Ecuadorian populations display low genetic and nucleotide diversity among sampled sites. Furthermore, indexes calculated for the population genetic analysis (Table 3), such as the overall value of the F_ST_’s (F_ST_ = 0.152), suggest that there is a low genetic differentiation among populations, similar to the detection of one population (K = 1) by Structure.

A similar number of haplotypes using the ND4 gene have been reported for Uruguay [40], Paraguay [41], and French Guiana [18] (2 haplotypes). Likewise, other countries in South America have reported a reduced number of haplotypes, between 4–6 haplotypes. This is the case of Venezuela with 6 haplotypes [42], Colombia with 4 haplotypes [15], Peru with 5 haplotypes [43], and Bolivia with 4 haplotypes [17]. Contrary to these results, New World populations of *Ae. aegypti* have reported a wide range of a number of haplotypes for the ND4 gene. In fact, the highest diversity was recorded in populations of the Southern United States and Mexico, with 25 haplotypes [21]. The second region with the highest number of haplotypes is Argentina with 20 haplotypes [41], followed by Brazil with 8-13 haplotypes [19,20]. This pattern of reduced genetic diversity is reported among New World populations of *Ae. aegypti* with the use of other molecular markers. For instance, studies that used microsatellites to determine genetic diversity have found allelic richness ranging from two to nine alleles within populations from Bolivia [17], Brazil, French Guiana [44], and Argentina [45]. In comparison, African populations present higher allelic diversity with allelic richness ranging from nine to 23 alleles [46,47]. Similarly, studies that genotyped for genome-wide SNPs have found three to four genetic groups in American [46,48,49,50], whereas 11 genetic clusters are found among African populations [46]. Since *Ae. aegypti* is an introduced species to the American continent, the genetic makeup of its populations represents a subset of the genetic diversity of African populations [4]. The diversity seen in each country is associated with the number of colonization and re-colonization events, the number of individuals introduced, and population expansion [51,52,53]. As well as, complex population dynamics associated with cycles of vectorial control [54,55]. Signals of population bottlenecks are reported for the Perú, Venezuela, Mexico, and Brazil populations, where the small number of haplotypes is associated with the effect of vectorial control [54]. This could also be the case for Ecuadorian populations of the present study, as further discussed below. Still, in some studies, the proportion of haplotypes detected is linked to the number of specimens analyzed and geographic representation of these specimens [18,41].

The phylogenetic analyses presented in this study include the ND4 haplotypes reported from around the world (Figure 3), as well as the comparative analysis of the percentage of identity of material deposited in the GenBank (Table 2), indicate that the Ecuadorian haplotype 1 is an exact match with haplotypes reported in Brazil [10,20], Mexico [21], Peru [16], Colombia [12,15], Bolivia [17], Senegal, Guinea, Ivory Coast, Nigeria, Cameroon [13], and Cape Verde [23]. Whereas, haplotype 2 was identical to haplotypes reported in Brazil [10,20], Peru [16], and Mexico [21]. These observations are in accordance with previous studies that indicate that the origin of New World populations in West Africa [4,56]. We presume that the genetic diversity of Ecuadorian populations are the result of either a few colonization events or cycles of local extinction and re-colonization by founder individuals, with less genetic diversity [57]. Evidence of population contraction is found through the analyses of neutrality tests (Table 3). The overall value of the Tajima D (3.98848, *p* > 0.001, Table 3) showed that Ecuadorian populations appear to have experienced a sudden population contraction and are undergoing balancing selection [58]. Even though individual values of each neutrality test were not significant, eight sites presented neutrality tests with a significant *p*-value (Table 3), suggesting that these populations are undergoing population contraction. This event is possibly associated with vectorial control, as reported in other South American countries [49]. Preliminary work from our group, analyzing a portion of the sampled material in this study by using the COI gene, detected a similar pattern of genetic diversity with two haplotypes being reported [59]. Thus, to have a complete understanding of the genetic diversity and population structure for this gene, there is a need to expand site representation and the number of individuals per site for COI molecular marker.

There is no clear record of the first introduction of *Ae. aegypti* in Ecuador. The most plausible hypothesis is that this vector arrived in the country associated with human activities during the late 18th century through the seaport city of Guayaquil [41]. Since then, *Ae. aegypti* has extended its distribution from the Pacific coast to the Amazon basin region, reaching locations at 1650 m of altitude at the northeastern mountain slopes (Cevallos, Unpublished data). In contrast, the introduction of this vector to the Galapagos Islands is well documented, *Ae. aegypti* was first recorded in Santa Cruz and San Cristobal Islands during 2001 [60].

Even though genetic differentiation among populations was low (FST = 0.152). There was some level of genetic differentiation among populations when individual values of the FST´s on a population by population basis were compared. This is the case of the sites Lita and Cumandá (FST = 0.56), and Lita and Machala (FST = 0.78). These localities show high levels of genetic differentiation, possibly associated with the distance between localities and geographical isolation, especially Lita located in a remote area of Ecuador. Most of the variation was found within the population, which is consistent with the levels of genetic connectivity across populations (Table 4). Furthermore, the highest number of migrants per generation was detected between Borbón and Nueva Loja (Nm = 23.74). Followed by Guayaquil and Santa Cruz (Nm = 4.93); together with Guayaquil and Cumandá (Nm = 4.63, Table 4). Genetic connectivity across populations is often associated with human-aided activities and natural dispersal processes [61,62]. We believe human activities have aided the dispersal in these populations since some sites are ports or places associated with the mass movement of people, which allows the vector to spread and maintain gene flow across populations.

Understanding the population dynamics and genetic structure of populations of *Ae. aegypti* in tropical and subtropical areas of Ecuador is of considerable importance, given the number of arboviruses this vector transmits [3]. The distributional patterns, genetic structure, and genetic diversity of the vector have implications towards understanding the epidemiology of arboviral diseases, vectorial control, and areas of high levels of genetic connectivity. All this information can help design more effective prevention and control strategies for areas with a high prevalence of vector-borne diseases.

## 5. Conclusions

Phylogenetic relationship analysis of Ecuadorian populations of *Ae. aegypti* showed two haplotypes. Nonetheless, these populations presented low genetic diversity and maintained genetic connectivity. The latter could be explained by passive dispersal patterns. Hence, vector genetic identity may help to design a more efficient approach when control programs are planned for localities with a high incidence of arboviral clinical cases.

## Figures and Tables

**Table 1:** Localities were sampled to collect *Aedes aegypti* individuals from three regions of Ecuador.

Region	N°	Locality	Longitude	Latitude	Collection Year	Sample Size
Amazon basin	1	Francisco de Orellana	−76.679	−0.468	2013	6
	2	Macas	−78.133	−2.323	2013	7
	3	Nueva Loja	−76.877	0.064	2013	9
	4	Puyo	−77.956	−1.479	2012	4
	5	Tena	−77.820	−0.982	2015	5
Galapagos Islands	6	Santa Cruz	−90.325	−0.715	2014	9
	7	San Cristobal	−89.594	−0.910	2014	3
Pacific coast	8	Babahoyo	−79.679	−1.787	2014	5
	9	Borbón	−78.987	1.093	2018	10
	10	Cumandá	−79.135	−2.208	2014	8
	11	Esmeraldas	−79.660	0.947	2014	5
	12	Guayaquil	−79.921	−2.246	2016	40
	13	Lita	−78.451	0.869	2018	9
	14	Machala	−79.927	−3.259	2017	8
	15	Manta	−80.732	0.955	2017	4
	16	Quinsaloma	−79.310	−1.204	2017	2
	17	Santo Domingo	−79.156	−0.222	2013	3
**Total**						137

**Table 2:** Ecuadorian haplotypes of the ND4 mitochondrial marker were identified in *Aedes aegypti* and compared to haplotypes found in the published literature. X refers to the absence of the haplotype.

Ecuadorian Haplotype	America, Asia, Africa	Cape Verde	Brazilian Amazon	Brazil	Mexico	Colombia	Peru	Bolivia
H1	H15	H6	H1	H11	H13	H2 [15]; H81 [12]	H2	H4
H2	H5	X	H10	H2	H20	X	H1	X

**Table 3:** Genetic diversity of the 17 *Aedes aegypti* populations from Ecuador. N refers to the number of individuals. Scheme 0. *p*-value: ^a^ *p* < 0.05; ^b^ *p* < 0.01; ^c^ *p* < 0.001.

Region	N°	Locality	N	h	π	S	D	F
Amazon basin	1	Fco. de Orellana	6	0.545 ± 0.062	0.01685 ± 0.00190	8	2.58449 ^b^	7.657 ^b^
	2	Macas	6	0.264 ± 0.136	0.00821 ± 0.00423	8	−0.60818	4.844
	3	Nueva Loja	9	0.366 ± 0.112	0.01105 ± 0.00339	8	0.89988	6.910 ^b^
	4	Puyo	4	0.000 ± 0.000	0.000 ± 0.000	0	0.000	0.000
	5	Tena	5	0.000 ± 0.000	0.000 ± 0.000	0	0.000	0.000
Galapagos Islands	6	Santa Cruz	9	0.523 ± 0.048	0.01609 ± 0.00147	8	2.77503 ^b^	8.980 ^b^
	7	San Cristobal	3	0.000 ± 0.000	0.000 ± 0.000	0	0.000	0.000
Pacific coast	8	Babahoyo	5	0.533 ± 0.095	0.01586 ± 0.00282	8	2.19508 ^a^	6.859 ^a^
	9	Borbón	10	0.505 ± 0.056	0.01470 ± 0.00163	8	2.67114 ^b^	9.158 ^b^
	10	Cumandá	8	0.500 ± 0.074	0.01498 ± 0.00222	8	3.98848 ^c^	18.142
	11	Esmeraldas	5	0.533 ± 0.095	0.01501 ± 0.00051	8	2.19508 ^a^	6.859 ^a^
	12	Guayaquil	40	0.425 ± 0.042	0.01309 ± 0.00621	8	2.78019 ^b^	12.641
	13	Lita	9	0.000 ± 0.000	0.000 ± 0.000	0	0.000	0.000
	14	Machala	8	0.233 ± 0.126	0.00697 ± 0.00375	8	−0.81386	4.641
	15	Manta	4	0.000 ± 0.000	0.000 ± 0.000	0	0.000	0.000
	16	Quinsaloma	2	0.000 ± 0.000	0.000 ± 0.000	0	0.000	0.000
	17	Santo Domingo	3	0.0296 ± 0.172	0.01569 ± 0.00725	8	1.28387	5.025
		**Total**	137	0.465 ± 0.016	0.01501 ± 0.00051	8	3.98848 ^c^	18.142

**Table 4:** Genetic distances (FST values; below the diagonal), and the number of migrants per generation (Nm; above the diagonal), among seven populations of *Aedes aegypti* from continental and insular regions of Ecuador. *p*-value: ^a^ *p* < 0.05.

Locality	Nueva Loja	Santa Cruz	Cumandá	Guayaquil	Machala	Borbón	Lita
Nueva Loja	-	2.57	1.59	0.00	0.37	24.73	2.33
Santa Cruz	0.07	-	0.00	4.93	2.36	0.00	0.44
Cumandá	0.16	−0.11	-	2.65	4.63	11.24	0.86
Guayaquil	−0.05	0.07	0.12	-	0.51	0.00	0.9
Machala	0.44	0.07	0.02	0.39	-	1.22	0.08
Borbón	−0.03	−0.05	−0.02	−0.04	0.27	-	0.86
Lita	0.10	0.41	0.56 ^a^	0.14	0.78 ^a^	0.32	-

**Table 5:** Results from the analysis of the molecular variances (AMOVA). Data were partitioned to test the effect of the region (Pacific coast, Amazon basin, and Galapagos Islands), as well as the effect of geographical regions.

No. Groups	Partitions	Test	Among Groups	Among Populations	Within Groups
3	(3) (6) (9,10,12,14)	Three partitions	−14.85	23.91	90.94
4	(3) (6) (10,12,14) (9,13)	Four partitions	−14.85	23.91	90.94
5	(3) (6) (10,12,14) (9) (13)	Five partitions	−14.85	23.91	90.94
6	(3) (6) (10) (12,14) (9) (13)	Geographical Barriers	−14.85	23.91	90.94
7	(3) (6) (10) (12) (14) (9) (13)	Geographical Barriers II	−2.8	17.99	84.8

## Data Availability

Sequences used in this study are depostied in Genbank under the accession numbers MK905895-MK905989, MK905991-MK906024, MW316314-MW316322.

## Supplementary Materials

The following are available online at https://www.mdpi.com/2075-4450/12/3/200/s1, Table S1: Haplotype designation for individuals of Aedes aegypti collected in 17 localities from three regions of Ecuador; Table S2: Reported sequences of Aedes aegypti for ND4 mitochondrial marker used for phylogenetic relationship analysis; Table S3: Reported haplotypes of Aedes aegypti from America and Africa using ND4 mitochondrial marker.
