# Supplementary material for: Two Haplotypes of Aedes aegypti Detected by ND4 Mitochondrial Marker in Three Regions of Ecuador
Source: Insects. 2021 Feb 27;12(3):200. doi: 10.3390/insects12030200 (PMC7996963; doi:10.3390/insects12030200)
Supplement: Supplementary file 1 [file insects-12-00200-s001.pdf]

Supplementary Table S1. Haplotype designation for individuals of *Aedes aegypti* collected in 17 localities from three regions of Ecuador

Accession N°	ID	Organism	Country	Locality	Haplotype	Reference
MK905962.1	ECU10793	*Aedes aegypti*	Ecuador	Guayaquil	H1	This study
MK905963.1	ECU10800	*Aedes aegypti*	Ecuador	Guayaquil	H1	This study
MK905964.1	ECU10804	*Aedes aegypti*	Ecuador	Guayaquil	H1	This study
MK905965.1	ECU10806	*Aedes aegypti*	Ecuador	Guayaquil	H1	This study
MK905966.1	ECU10813	*Aedes aegypti*	Ecuador	Guayaquil	H2	This study
MK905967.1	ECU10845	*Aedes aegypti*	Ecuador	Guayaquil	H2	This study
MK905968.1	ECU10853	*Aedes aegypti*	Ecuador	Guayaquil	H1	This study
MK905969.1	ECU10857	*Aedes aegypti*	Ecuador	Guayaquil	H1	This study
MK905970.1	ECU10863	*Aedes aegypti*	Ecuador	Guayaquil	H1	This study
MK905971.1	ECU10874	*Aedes aegypti*	Ecuador	Guayaquil	H1	This study
MK905972.1	ECU10877	*Aedes aegypti*	Ecuador	Guayaquil	H1	This study
MK905973.1	ECU10881	*Aedes aegypti*	Ecuador	Guayaquil	H1	This study
MK905974.1	ECU10882	*Aedes aegypti*	Ecuador	Guayaquil	H1	This study
MK905975.1	ECU10884	*Aedes aegypti*	Ecuador	Guayaquil	H2	This study
MK905976.1	ECU10887	*Aedes aegypti*	Ecuador	Guayaquil	H2	This study
MK905977.1	ECU10889	*Aedes aegypti*	Ecuador	Guayaquil	H1	This study
MK905978.1	ECU10891	*Aedes aegypti*	Ecuador	Guayaquil	H1	This study
MK905979.1	ECU10893	*Aedes aegypti*	Ecuador	Guayaquil	H2	This study
MK905980.1	ECU10896	*Aedes aegypti*	Ecuador	Guayaquil	H1	This study
MK905981.1	ECU10899	*Aedes aegypti*	Ecuador	Guayaquil	H2	This study
MK905982.1	ECU10901	*Aedes aegypti*	Ecuador	Guayaquil	H1	This study
MK905983.1	ECU10902	*Aedes aegypti*	Ecuador	Guayaquil	H1	This study
MK905984.1	ECU10904	*Aedes aegypti*	Ecuador	Guayaquil	H1	This study
MK905985.1	ECU10915	*Aedes aegypti*	Ecuador	Guayaquil	H1	This study
MK905986.1	ECU10916	*Aedes aegypti*	Ecuador	Guayaquil	H1	This study
MK905987.1	ECU10917	*Aedes aegypti*	Ecuador	Guayaquil	H2	This study
MK905988.1	ECU10918	*Aedes aegypti*	Ecuador	Guayaquil	H1	This study
MK905989.1	ECU20715	*Aedes aegypti*	Ecuador	Guayaquil	H2	This study
MK905991.1	ECU20723	*Aedes aegypti*	Ecuador	Quinsaloma	H1	This study
MK905992.1	ECU20725	*Aedes aegypti*	Ecuador	Quinsaloma	H1	This study
MK905993.1	ECU20727	*Aedes aegypti*	Ecuador	Machala	H2	This study
MK905994.1	ECU20728	*Aedes aegypti*	Ecuador	Machala	H2	This study
MK905995.1	ECU20729	*Aedes aegypti*	Ecuador	Machala	H1	This study
MK905996.1	ECU20738	*Aedes aegypti*	Ecuador	Guayaquil	H1	This study
MK905998.1	ECU20746	*Aedes aegypti*	Ecuador	Nueva Loja	H1	This study
MK905999.1	ECU20748	*Aedes aegypti*	Ecuador	Guayaquil	H1	This study
MK906000.1	ECU20749	*Aedes aegypti*	Ecuador	Manta	H1	This study
MK906001.1	ECU20754	*Aedes aegypti*	Ecuador	Cumandá	H1	This study
MK906002.1	ECU20755	*Aedes aegypti*	Ecuador	Cumandá	H2	This study

MK906003.1	ECU20756	*Aedes aegypti*	Ecuador	Lita	H1	This study
MK906004.1	ECU20763	*Aedes aegypti*	Ecuador	Santa Cruz	H1	This study
MK906004.1	ECU20764	*Aedes aegypti*	Ecuador	Santa Cruz	H2	This study
MW316322	ECU20772	*Aedes aegypti*	Ecuador	Tena	H1	This study
MK906006.1	ECU20773	*Aedes aegypti*	Ecuador	Tena	H1	This study
MW316314	ECU20775	*Aedes aegypti*	Ecuador	Macas	H1	This study
MK906007.1	ECU20779	*Aedes aegypti*	Ecuador	Francisco de Orellana	H1	This study
MK906008.1	ECU22024	*Aedes aegypti*	Ecuador	Lita	H1	This study
MK906009.1	ECU22025	*Aedes aegypti*	Ecuador	Lita	H1	This study
MK906010.1	ECU22027	*Aedes aegypti*	Ecuador	Lita	H1	This study
MK906011.1	ECU22028	*Aedes aegypti*	Ecuador	Lita	H1	This study
MK906012.1	ECU22029	*Aedes aegypti*	Ecuador	Lita	H1	This study
MK906013.1	ECU22030	*Aedes aegypti*	Ecuador	Lita	H1	This study
MK906014.1	ECU22032	*Aedes aegypti*	Ecuador	Lita	H1	This study
MK906015.1	ECU22347	*Aedes aegypti*	Ecuador	Borbón	H2	This study
MK906016.1	ECU22349	*Aedes aegypti*	Ecuador	Borbón	H2	This study
MK906017.1	ECU22350	*Aedes aegypti*	Ecuador	Borbón	H2	This study
MK906018.1	ECU22353	*Aedes aegypti*	Ecuador	Borbón	H2	This study
MK906019.1	ECU22354	*Aedes aegypti*	Ecuador	Borbón	H1	This study
MK906020.1	ECU22355	*Aedes aegypti*	Ecuador	Borbón	H1	This study
MK906021.1	ECU22357	*Aedes aegypti*	Ecuador	Borbón	H1	This study
MK906022.1	ECU22358	*Aedes aegypti*	Ecuador	Borbón	H1	This study
MK906023.1	ECU22359	*Aedes aegypti*	Ecuador	Borbón	H1	This study
MK906024.1	ECU22360	*Aedes aegypti*	Ecuador	Borbón	H1	This study
MK905895.1	ECU7922	*Aedes aegypti*	Ecuador	Guayaquil	H2	This study
MK905896.1	ECU7938	*Aedes aegypti*	Ecuador	Guayaquil	H1	This study
MK905897.1	ECU7939	*Aedes aegypti*	Ecuador	Guayaquil	H1	This study
MW316319	ECU7940	*Aedes aegypti*	Ecuador	Guayaquil	H1	This study
MK905898.1	ECU7964	*Aedes aegypti*	Ecuador	Manta	H1	This study
MK905899.1	ECU8020	*Aedes aegypti*	Ecuador	Guayaquil	H1	This study
MK905900.1	ECU8021	*Aedes aegypti*	Ecuador	Guayaquil	H1	This study
MW316315	ECU8023	*Aedes aegypti*	Ecuador	Puyo	H1	This study
MK905901.1	ECU8024	*Aedes aegypti*	Ecuador	Puyo	H1	This study
MK905902.1	ECU8025	*Aedes aegypti*	Ecuador	Manta	H1	This study
MK905903.1	ECU8026	*Aedes aegypti*	Ecuador	Manta	H1	This study
MK905904.1	ECU8027	*Aedes aegypti*	Ecuador	Santo Domingo	H1	This study
MK905905.1	ECU8029	*Aedes aegypti*	Ecuador	Tena	H1	This study
MW316316	ECU8033	*Aedes aegypti*	Ecuador	Nueva Loja	H1	This study
MK905906.1	ECU8034	*Aedes aegypti*	Ecuador	Nueva Loja	H1	This study
MK905907.1	ECU8044	*Aedes aegypti*	Ecuador	Guayaquil	H1	This study
MK905908.1	ECU8168	*Aedes aegypti*	Ecuador	Guayaquil	H1	This study
MK905909.1	ECU8177	*Aedes aegypti*	Ecuador	Guayaquil	H2	This study
MK905910.1	ECU8247	*Aedes aegypti*	Ecuador	Nueva Loja	H1	This study

MW316317	ECU8248	*Aedes aegypti*	Ecuador	Santa Cruz	H1	This study
MK905911.1	ECU8299	*Aedes aegypti*	Ecuador	Santa Cruz	H1	This study
MK905912.1	ECU8300	*Aedes aegypti*	Ecuador	Santa Cruz	H1	This study
MK905913.1	ECU8306	*Aedes aegypti*	Ecuador	Santa Cruz	H2	This study
MK905914.1	ECU8307	*Aedes aegypti*	Ecuador	Santa Cruz	H2	This study
MK905915.1	ECU8700	*Aedes aegypti*	Ecuador	Macas	H2	This study
MK905916.1	ECU8701	*Aedes aegypti*	Ecuador	Macas	H2	This study
MK905917.1	ECU8702	*Aedes aegypti*	Ecuador	Macas	H2	This study
MK905918.1	ECU8704	*Aedes aegypti*	Ecuador	Macas	H2	This study
MK905919.1	ECU8705	*Aedes aegypti*	Ecuador	Macas	H2	This study
MK905920.1	ECU8706	*Aedes aegypti*	Ecuador	Macas	H2	This study
MK905921.1	ECU8768	*Aedes aegypti*	Ecuador	Nueva Loja	H1	This study
MK905922.1	ECU8769	*Aedes aegypti*	Ecuador	Nueva Loja	H1	This study
MK905923.1	ECU8790	*Aedes aegypti*	Ecuador	Nueva Loja	H2	This study
MW316320	ECU8889	*Aedes aegypti*	Ecuador	Tena	H1	This study
MK905924.1	ECU8890	*Aedes aegypti*	Ecuador	Tena	H1	This study
MK905925.1	ECU8975	*Aedes aegypti*	Ecuador	Cumandá	H2	This study
MK905926.1	ECU8976	*Aedes aegypti*	Ecuador	Cumandá	H2	This study
MK905927.1	ECU8977	*Aedes aegypti*	Ecuador	Cumandá	H2	This study
MK905928.1	ECU8978	*Aedes aegypti*	Ecuador	Cumandá	H1	This study
MK905929.1	ECU8979	*Aedes aegypti*	Ecuador	Cumandá	H1	This study
MK905930.1	ECU8998	*Aedes aegypti*	Ecuador	Machala	H2	This study
MK905931.1	ECU8999	*Aedes aegypti*	Ecuador	Machala	H2	This study
MK905932.1	ECU9000	*Aedes aegypti*	Ecuador	Machala	H2	This study
MK905933.1	ECU9003	*Aedes aegypti*	Ecuador	Machala	H2	This study
MK905934.1	ECU9010	*Aedes aegypti*	Ecuador	Machala	H2	This study
MK905935.1	ECU9060	*Aedes aegypti*	Ecuador	Francisco de Orellana	H2	This study
MK905936.1	ECU9061	*Aedes aegypti*	Ecuador	Francisco de Orellana	H1	This study
MK905937.1	ECU9062	*Aedes aegypti*	Ecuador	Francisco de Orellana	H2	This study
MK905938.1	ECU9063	*Aedes aegypti*	Ecuador	Francisco de Orellana	H1	This study
MK905939.1	ECU9065	*Aedes aegypti*	Ecuador	Lita	H1	This study
MK905940.1	ECU9144	*Aedes aegypti*	Ecuador	Guayaquil	H2	This study
MK905941.1	ECU9170	*Aedes aegypti*	Ecuador	Babahoyo	H2	This study
MK905942.1	ECU9171	*Aedes aegypti*	Ecuador	Babahoyo	H2	This study
MK905943.1	ECU9172	*Aedes aegypti*	Ecuador	Babahoyo	H1	This study
MK905944.1	ECU9173	*Aedes aegypti*	Ecuador	Babahoyo	H1	This study
MK905945.1	ECU9175	*Aedes aegypti*	Ecuador	Babahoyo	H1	This study
MW316318	ECU9232	*Aedes aegypti*	Ecuador	Francisco de Orellana	H2	This study
MK905946.1	ECU9242	*Aedes aegypti*	Ecuador	Esmeraldas	H2	This study
MK905947.1	ECU9243	*Aedes aegypti*	Ecuador	Esmeraldas	H1	This study
MK905948.1	ECU9244	*Aedes aegypti*	Ecuador	Esmeraldas	H1	This study
MK905949.1	ECU9245	*Aedes aegypti*	Ecuador	Esmeraldas	H2	This study
MK905950.1	ECU9251	*Aedes aegypti*	Ecuador	Esmeraldas	H2	This study

MK905951.1	ECU9307	*Aedes aegypti*	Ecuador	Santa Cruz	H2	This study
MK905952.1	ECU9308	*Aedes aegypti*	Ecuador	Santa Cruz	H2	This study
MK905953.1	ECU9332	*Aedes aegypti*	Ecuador	San Cristobal	H1	This study
MK905954.1	ECU9333	*Aedes aegypti*	Ecuador	San Cristobal	H1	This study
MK905955.1	ECU9337	*Aedes aegypti*	Ecuador	San Cristobal	H1	This study
MW316321	ECU9484	*Aedes aegypti*	Ecuador	Cumandá	H2	This study
MK905956.1	ECU9491	*Aedes aegypti*	Ecuador	Nueva Loja	H2	This study
MK905957.1	ECU9492	*Aedes aegypti*	Ecuador	Nueva Loja	H1	This study
MK905958.1	ECU9500	*Aedes aegypti*	Ecuador	Puyo	H1	This study
MK905959.1	ECU9501	*Aedes aegypti*	Ecuador	Puyo	H1	This study
MK905960.1	ECU9521	*Aedes aegypti*	Ecuador	Santo Domingo	H1	This study
MK905961.1	ECU9522	*Aedes aegypti*	Ecuador	Santo Domingo	H2	This study

Supplementary Table 2. Reported sequences of *Aedes aegypti* for ND4 mitochondrial marker used for phylogenetic relationship analysis

Accession N°	ID	Organism	Locality	Reference
GU188856.2		*Culex quinquefasciatus*		Behura et al., 2011
JQ926708.1	Haplotype 4	*Aedes aegypti*	Bolivia	Paupy et al., 2012
EF153748.1	Haplotype 5	*Aedes aegypti*	Brazil	Costa et al., unpublished
EF153756.1	Haplotype 13	*Aedes aegypti*	Brazil	Costa et al., unpublished
EF153758.1	Haplotype 15	*Aedes aegypti*	Brazil	Costa et al., unpublished
EF153760.1	Haplotype 17	*Aedes aegypti*	Brazil	Costa et al., unpublished
KF922335.1	Haplotype 3	*Aedes aegypti*	Brazil	Fraga et al., 2013
EU650408.1	Haplotype 4	*Aedes aegypti*	Brazil	Lima and Scarpassa, 2009
EU650413.1	Haplotype 9	*Aedes aegypti*	Brazil	Lima and Scarpassa, 2009
AY906835.1	Clone 1	*Aedes aegypti*	Brazil	Paduan and Ribolla, 2008
AY906838.1	Clone 4	*Aedes aegypti*	Brazil	Paduan and Ribolla, 2008
AY906840.1	Clone 6	*Aedes aegypti*	Brazil	Paduan and Ribolla, 2008
AY906845.1	Clone 11	*Aedes aegypti*	Brazil	Paduan and Ribolla, 2008
AY906850.1	Clone 16	*Aedes aegypti*	Brazil	Paduan and Ribolla, 2008
MH382099.1	Haplotype 8	*Aedes aegypti*	Brazil	Paiva et al., unpublished
EF562501.1	Haplotype 1	*Aedes aegypti*	Cameroon	Paupy et al., Unpublished
KR053060.1	ISP:11_S40_3	*Aedes aegypti*	Chile	Nunez et al., unpublished
KM203317.1	Haplotype 69	*Aedes aegypti*	Colombia	Jaimes-Duenez et al., 2015
KM203321.1	Haplotype 73	*Aedes aegypti*	Colombia	Jaimes-Duenez et al., 2015
KM203322.1	Haplotype 74	*Aedes aegypti*	Colombia	Jaimes-Duenez et al., 2015
KM203323.1	Haplotype 75	*Aedes aegypti*	Colombia	Jaimes-Duenez et al., 2015
KM203325.1	Haplotype 77	*Aedes aegypti*	Colombia	Jaimes-Duenez et al., 2015
KM203328.1	Haplotype 80	*Aedes aegypti*	Colombia	Jaimes-Duenez et al., 2015
KM203330.1	Haplotype82	*Aedes aegypti*	Colombia	Jaimes-Duenez et al., 2015
KM203332.1	Haplotype 84	*Aedes aegypti*	Colombia	Jaimes-Duenez et al., 2015
KM042180.1	COLMVS1L8	*Aedes aegypti*	Colombia	Jaramillo et al,, unpublished
KM042189.1	COLAP10L326	*Aedes aegypti*	Colombia	Jaramillo et al,, unpublished
KM042195.1	COLCPC1L19	*Aedes aegypti*	Colombia	Jaramillo et al,, unpublished
KM042196.1	COLCPP2L29	*Aedes aegypti*	Colombia	Jaramillo et al,, unpublished
KM042199.1	COLCHO1L72	*Aedes aegypti*	Colombia	Jaramillo et al,, unpublished
JQ926709.1	RCI 1	*Aedes aegypti*	Cote d'Ivoire	Paupy et al., 2012
JQ926717.1	Guinea 1	*Aedes aegypti*	Guinea	Paupy et al., 2012
JQ926711.1	Martinique 1	*Aedes aegypti*	Martinique	Paupy et al., 2012
JQ926713.1	Mexico 1	*Aedes aegypti*	Mexico	Paupy et al., 2012
JQ926714.1	MEX4	*Aedes aegypti*	Mexico	Paupy et al., 2012
JX297257.1	GUY31	*Aedes aegypti*	Mexico, Sonora, Guaymas	Pfeiler et al., unpublished
JX297251.1	HER1	*Aedes aegypti*	Mexico, Sonora, Hermosillo	Pfeiler et al., unpublished

FJ428769.1	M1_20L	*Aedes aegypti*	Myanmar	Hlaing et al., 2009
KC800691.1	Isolate 4	*Aedes aegypti*	Nigeria	Ayorinde et al., 2013 (Direct submission)
DQ177154.1	Piura 1	*Aedes aegypti*	Peru, Piura	da Costa-da-Silva et al., 2005
DQ177155.1	Piura 2	*Aedes aegypti*	Peru, Piura	da Costa-da-Silva et al., 2005
JX427511.1	Fg1057	*Aedes aegypti*	Senegal, Fongolimbi	Moore et al., 2013
FJ428796.1	T5_12L	*Aedes aegypti*	Thailand	Hlaing et al., 2009
JX427505.1	ThaiHap2	*Aedes aegypti*	Thailand	Moore et al., 2013
KU207146.1	HNL	*Aedes aegypti*	USA	Hasty et al., 2015
JX297250.1	TUC11	*Aedes aegypti*	USA, Arizona, Tucson	Pfeiler et al., unpublished

Supplementary Table 3. Reported haplotypes of Aedes *aegypti* from America and Africa using ND4 mitochondrial marker

Accession N°	ID	Organism	Locality	Reference
KX580042.1	COLSM1L1330	*Aedes aegypti*	Colombia	Atencia et al., 2018
KX580043.1	COLSM1L1559	*Aedes aegypti*	Colombia	Atencia et al., 2018
KX580044.1	COLSM3L1258	*Aedes aegypti*	Colombia	Atencia et al., 2018
KX580045.1	COLSF1L2128	*Aedes aegypti*	Colombia	Atencia et al., 2018
DQ176828.2	Haplotype 01	*Aedes aegypti*	America	Bracco et al., 2007
DQ176829.2	Haplotype 02	*Aedes aegypti*	America	Bracco et al., 2007
DQ176830.2	Haplotype 03	*Aedes aegypti*	America	Bracco et al., 2007
DQ176831.2	Haplotype 05	*Aedes aegypti*	America	Bracco et al., 2007
DQ176833.2	Haplotype 07	*Aedes aegypti*	Africa	Bracco et al., 2007
DQ176834.2	Haplotype 08	*Aedes aegypti*	Africa	Bracco et al., 2007
DQ176835.2	Haplotype 09	*Aedes aegypti*	Africa	Bracco et al., 2007
DQ176836.2	Haplotype 10	*Aedes aegypti*	Africa	Bracco et al., 2007
DQ176837.2	Haplotype 11	*Aedes aegypti*	Africa	Bracco et al., 2007
DQ176838.2	Haplotype 12	*Aedes aegypti*	Africa	Bracco et al., 2007
DQ176839.2	Haplotype 13	*Aedes aegypti*	Africa	Bracco et al., 2007
DQ176840.2	Haplotype 14	*Aedes aegypti*	Africa	Bracco et al., 2007
DQ176841.2	Haplotype 15	*Aedes aegypti*	Africa	Bracco et al., 2007
DQ176842.2	Haplotype 16	*Aedes aegypti*	Africa	Bracco et al., 2007
DQ176843.2	Haplotype 17	*Aedes aegypti*	Africa	Bracco et al., 2007
DQ176845.2	Haplotype 19	*Aedes aegypti*	Asia	Bracco et al., 2007
DQ176846.2	Haplotype 20	*Aedes aegypti*	Asia	Bracco et al., 2007
DQ176847.2	Haplotype 21	*Aedes aegypti*	Asia	Bracco et al., 2007
DQ176848.2	Haplotype 22	*Aedes aegypti*	Asia	Bracco et al., 2007
DQ176849.2	Haplotype 23	*Aedes aegypti*	Asia	Bracco et al., 2007
MT721877.1	C09	*Aedes aegypti*	Cape Verde	Campos et al., 2020
MT721878.1	G06	*Aedes aegypti*	Cape Verde	Campos et al., 2020
MT721879.1	G02	*Aedes aegypti*	Cape Verde	Campos et al., 2020
MT721880.1	C12	*Aedes aegypti*	Cape Verde	Campos et al., 2020
MT721881.1	A09	*Aedes aegypti*	Cape Verde	Campos et al., 2020
MT721882.1	D01	*Aedes aegypti*	Cape Verde	Campos et al., 2020
MT721883.1	E01	*Aedes aegypti*	Cape Verde	Campos et al., 2020
MT721884.1	H07	*Aedes aegypti*	Cape Verde	Campos et al., 2020
MT721885.1	F07	*Aedes aegypti*	Cape Verde	Campos et al., 2020
MT721886.1	E08	*Aedes aegypti*	Cape Verde	Campos et al., 2020
MT721887.1	E07	*Aedes aegypti*	Cape Verde	Campos et al., 2020
MT721888.1	B08	*Aedes aegypti*	Cape Verde	Campos et al., 2020
MT721889.1	B11	*Aedes aegypti*	Cape Verde	Campos et al., 2020
MT721890.1	H08	*Aedes aegypti*	Cape Verde	Campos et al., 2020
MT721891.1	F01	*Aedes aegypti*	Cape Verde	Campos et al., 2020

MT721892.1	E11	*Aedes aegypti*	Cape Verde	Campos et al., 2020
MT721893.1	B02	*Aedes aegypti*	Cape Verde	Campos et al., 2020
MT721894.1	D04	*Aedes aegypti*	Cape Verde	Campos et al., 2020
MT721895.1	G03	*Aedes aegypti*	Cape Verde	Campos et al., 2020
MT721896.1	D02	*Aedes aegypti*	Cape Verde	Campos et al., 2020
MT721897.1	A04	*Aedes aegypti*	Cape Verde	Campos et al., 2020
MT721898.1	F09	*Aedes aegypti*	Cape Verde	Campos et al., 2020
MT721899.1	G05	*Aedes aegypti*	Cape Verde	Campos et al., 2020
MT721900.1	H01	*Aedes aegypti*	Cape Verde	Campos et al., 2020
MT721901.1	H04	*Aedes aegypti*	Cape Verde	Campos et al., 2020
MT721902.1	F06	*Aedes aegypti*	Cape Verde	Campos et al., 2020
MT721903.1	D08	*Aedes aegypti*	Cape Verde	Campos et al., 2020
MT721904.1	G01	*Aedes aegypti*	Cape Verde	Campos et al., 2020
MT721905.1	A03	*Aedes aegypti*	Cape Verde	Campos et al., 2020
MT721906.1	A11	*Aedes aegypti*	Cape Verde	Campos et al., 2020
MT721907.1	E12	*Aedes aegypti*	Cape Verde	Campos et al., 2020
MT721908.1	D09	*Aedes aegypti*	Cape Verde	Campos et al., 2020
MT721909.1	B10	*Aedes aegypti*	Cape Verde	Campos et al., 2020
MT721910.1	H12	*Aedes aegypti*	Cape Verde	Campos et al., 2020
MT721911.1	H09	*Aedes aegypti*	Cape Verde	Campos et al., 2020
MT721912.1	H06	*Aedes aegypti*	Cape Verde	Campos et al., 2020
MT721913.1	A08	*Aedes aegypti*	Cape Verde	Campos et al., 2020
MT721914.1	G12	*Aedes aegypti*	Cape Verde	Campos et al., 2020
MT721915.1	G11	*Aedes aegypti*	Cape Verde	Campos et al., 2020
MT721916.1	G10	*Aedes aegypti*	Cape Verde	Campos et al., 2020
MT721917.1	A07	*Aedes aegypti*	Cape Verde	Campos et al., 2020
MT721918.1	G07	*Aedes aegypti*	Cape Verde	Campos et al., 2020
MT721919.1	D12	*Aedes aegypti*	Cape Verde	Campos et al., 2020
MT721920.1	C01	*Aedes aegypti*	Cape Verde	Campos et al., 2020
MT721921.1	B03	*Aedes aegypti*	Cape Verde	Campos et al., 2020
MT721922.1	A02	*Aedes aegypti*	Cape Verde	Campos et al., 2020
MT721923.1	C11	*Aedes aegypti*	Cape Verde	Campos et al., 2020
MT721924.1	C02	*Aedes aegypti*	Cape Verde	Campos et al., 2020
MT721925.1	B06	*Aedes aegypti*	Cape Verde	Campos et al., 2020
MT721926.1	C06	*Aedes aegypti*	Cape Verde	Campos et al., 2020
MT721927.1	G04	*Aedes aegypti*	Cape Verde	Campos et al., 2020
MT721928.1	F05	*Aedes aegypti*	Cape Verde	Campos et al., 2020
MT721929.1	A06	*Aedes aegypti*	Cape Verde	Campos et al., 2020
MT721930.1	F11	*Aedes aegypti*	Cape Verde	Campos et al., 2020
MT721931.1	D10	*Aedes aegypti*	Cape Verde	Campos et al., 2020
MT721932.1	D11	*Aedes aegypti*	Cape Verde	Campos et al., 2020
MT721933.1	E10	*Aedes aegypti*	Cape Verde	Campos et al., 2020
MT721934.1	F02	*Aedes aegypti*	Cape Verde	Campos et al., 2020

MT721935.1	G08	*Aedes aegypti*	Cape Verde	Campos et al., 2020
MT721936.1	H11	*Aedes aegypti*	Cape Verde	Campos et al., 2020
MT721937.1	E06	*Aedes aegypti*	Cape Verde	Campos et al., 2020
MT721938.1	C07	*Aedes aegypti*	Cape Verde	Campos et al., 2020
MT721939.1	E04	*Aedes aegypti*	Cape Verde	Campos et al., 2020
MT721940.1	C04	*Aedes aegypti*	Cape Verde	Campos et al., 2020
MT721941.1	H03	*Aedes aegypti*	Cape Verde	Campos et al., 2020
MT721942.1	E05	*Aedes aegypti*	Cape Verde	Campos et al., 2020
MT721943.1	E03	*Aedes aegypti*	Cape Verde	Campos et al., 2020
MT721944.1	F03	*Aedes aegypti*	Cape Verde	Campos et al., 2020
MT721945.1	E09	*Aedes aegypti*	Cape Verde	Campos et al., 2020
MT721946.1	F08	*Aedes aegypti*	Cape Verde	Campos et al., 2020
MT721947.1	F12	*Aedes aegypti*	Cape Verde	Campos et al., 2020
MT721948.1	B12	*Aedes aegypti*	Cape Verde	Campos et al., 2020
MT721949.1	A10	*Aedes aegypti*	Cape Verde	Campos et al., 2020
MT721950.1	B01	*Aedes aegypti*	Cape Verde	Campos et al., 2020
MT721951.1	A05	*Aedes aegypti*	Cape Verde	Campos et al., 2020
MT721952.1	D05	*Aedes aegypti*	Cape Verde	Campos et al., 2020
MT721953.1	B04	*Aedes aegypti*	Cape Verde	Campos et al., 2020
MT721954.1	F04	*Aedes aegypti*	Cape Verde	Campos et al., 2020
MT721955.1	D03	*Aedes aegypti*	Cape Verde	Campos et al., 2020
MT721956.1	E02	*Aedes aegypti*	Cape Verde	Campos et al., 2020
MT721957.1	H05	*Aedes aegypti*	Cape Verde	Campos et al., 2020
MT721958.1	D06	*Aedes aegypti*	Cape Verde	Campos et al., 2020
MT721959.1	C10	*Aedes aegypti*	Cape Verde	Campos et al., 2020
MT721960.1	C08	*Aedes aegypti*	Cape Verde	Campos et al., 2020
MT721961.1	B07	*Aedes aegypti*	Cape Verde	Campos et al., 2020
KR349202.1	Haplotype A	*Aedes aegypti*	Venezuela	Carrozza et al., 2016
KR349203.1	Haplotype B	*Aedes aegypti*	Venezuela	Carrozza et al., 2016
KR349204.1	Haplotype C	*Aedes aegypti*	Venezuela	Carrozza et al., 2016
KR349205.1	Haplotype D	*Aedes aegypti*	Venezuela	Carrozza et al., 2016
KR349206.1	Haplotype E	*Aedes aegypti*	Venezuela	Carrozza et al., 2016
KR349207.1	Haplotype F	*Aedes aegypti*	Venezuela	Carrozza et al., 2016
KR349208.1	Haplotype G	*Aedes aegypti*	Venezuela	Carrozza et al., 2016
KR349209.1	Haplotype H	*Aedes aegypti*	Venezuela	Carrozza et al., 2016
KR349210.1	Haplotype I	*Aedes aegypti*	Venezuela	Carrozza et al., 2016
KR349211.1	Haplotype J	*Aedes aegypti*	Venezuela	Carrozza et al., 2016
KR349212.1	Haplotype K	*Aedes aegypti*	Venezuela	Carrozza et al., 2016
KR349213.1	Haplotype L	*Aedes aegypti*	Venezuela	Carrozza et al., 2016
DQ177153.1	Lima	*Aedes aegypti*	Peru - Lima	da Costa-da-Silva et al., 2005
DQ177154.1	Piura 1	*Aedes aegypti*	Peru - Piura	da Costa-da-Silva et al., 2005
DQ177155.1	Piura 2	*Aedes aegypti*	Peru - Piura	da Costa-da-Silva et al., 2005
AF334841.1	Isolate 1	*Aedes aegypti*	Mexico	Gorrochotegui-Escalante et al., 2002

AF334842.1	Isolate 2	*Aedes aegypti*	Mexico	Gorrochotegui-Escalante et al., 2002
AF334843.1	Isolate 3	*Aedes aegypti*	Mexico	Gorrochotegui-Escalante et al., 2002
AF334844.1	Isolate 4	*Aedes aegypti*	Mexico	Gorrochotegui-Escalante et al., 2002
AF334845.1	Isolate 5	*Aedes aegypti*	Mexico	Gorrochotegui-Escalante et al., 2002
AF334846.1	Isolate 6	*Aedes aegypti*	Mexico	Gorrochotegui-Escalante et al., 2002
AF334847.1	Isolate 7	*Aedes aegypti*	Mexico	Gorrochotegui-Escalante et al., 2002
AF334848.1	Isolate 8	*Aedes aegypti*	Mexico	Gorrochotegui-Escalante et al., 2002
AF334849.1	Isolate 9	*Aedes aegypti*	Mexico	Gorrochotegui-Escalante et al., 2002
AF334850.1	Isolate 10	*Aedes aegypti*	Mexico	Gorrochotegui-Escalante et al., 2002
AF334851.1	Isolate 11	*Aedes aegypti*	Mexico	Gorrochotegui-Escalante et al., 2002
AF334852.1	Isolate 12	*Aedes aegypti*	Mexico	Gorrochotegui-Escalante et al., 2002
AF334853.1	Isolate 13	*Aedes aegypti*	Mexico	Gorrochotegui-Escalante et al., 2002
AF334854.1	Isolate 14	*Aedes aegypti*	Mexico	Gorrochotegui-Escalante et al., 2002
AF334855.1	Isolate 15	*Aedes aegypti*	Mexico	Gorrochotegui-Escalante et al., 2002
AF334856.1	Isolate 16	*Aedes aegypti*	Mexico	Gorrochotegui-Escalante et al., 2002
AF334857.1	Isolate 17	*Aedes aegypti*	Mexico	Gorrochotegui-Escalante et al., 2002
AF334858.1	Isolate 18	*Aedes aegypti*	Mexico	Gorrochotegui-Escalante et al., 2002
AF334859.1	Isolate 19	*Aedes aegypti*	Mexico	Gorrochotegui-Escalante et al., 2002
AF334860.1	Isolate 20	*Aedes aegypti*	Mexico	Gorrochotegui-Escalante et al., 2002
AF334861.1	Isolate 21	*Aedes aegypti*	Mexico	Gorrochotegui-Escalante et al., 2002
AF334862.1	Isolate 22	*Aedes aegypti*	Mexico	Gorrochotegui-Escalante et al., 2002
AF334863.1	Isolate 23	*Aedes aegypti*	Mexico	Gorrochotegui-Escalante et al., 2002
AF334864.1	Isolate 24	*Aedes aegypti*	Mexico	Gorrochotegui-Escalante et al., 2002
AF334865.1	Isolate 25	*Aedes aegypti*	Mexico	Gorrochotegui-Escalante et al., 2002
KM203249.1	Haplotype 1	*Aedes aegypti*	Colombia	Jaimes-Duenez et al., 2015
KM203250.1	Haplotype 2	*Aedes aegypti*	Colombia	Jaimes-Duenez et al., 2015
KM203251.1	Haplotype 3	*Aedes aegypti*	Colombia	Jaimes-Duenez et al., 2015
KM203252.1	Haplotype 4	*Aedes aegypti*	Colombia	Jaimes-Duenez et al., 2015
KM203253.1	Haplotype 5	*Aedes aegypti*	Colombia	Jaimes-Duenez et al., 2015
KM203254.1	Haplotype 6	*Aedes aegypti*	Colombia	Jaimes-Duenez et al., 2015
KM203255.1	Haplotype 7	*Aedes aegypti*	Colombia	Jaimes-Duenez et al., 2015
KM203256.1	Haplotype 8	*Aedes aegypti*	Colombia	Jaimes-Duenez et al., 2015
KM203257.1	Haplotype 9	*Aedes aegypti*	Colombia	Jaimes-Duenez et al., 2015
KM203258.1	Haplotype 10	*Aedes aegypti*	Colombia	Jaimes-Duenez et al., 2015
KM203259.1	Haplotype 11	*Aedes aegypti*	Colombia	Jaimes-Duenez et al., 2015
KM203260.1	Haplotype 12	*Aedes aegypti*	Colombia	Jaimes-Duenez et al., 2015
KM203261.1	Haplotype 13	*Aedes aegypti*	Colombia	Jaimes-Duenez et al., 2015
KM203262.1	Haplotype 14	*Aedes aegypti*	Colombia	Jaimes-Duenez et al., 2015
KM203263.1	Haplotype 15	*Aedes aegypti*	Colombia	Jaimes-Duenez et al., 2015
KM203264.1	Haplotype 16	*Aedes aegypti*	Colombia	Jaimes-Duenez et al., 2015
KM203265.1	Haplotype 17	*Aedes aegypti*	Colombia	Jaimes-Duenez et al., 2015
KM203266.1	Haplotype 18	*Aedes aegypti*	Colombia	Jaimes-Duenez et al., 2015
KM203267.1	Haplotype 19	*Aedes aegypti*	Colombia	Jaimes-Duenez et al., 2015

KM203268.1	Haplotype 20	*Aedes aegypti*	Colombia	Jaimes-Duenez et al., 2015
KM203269.1	Haplotype 21	*Aedes aegypti*	Colombia	Jaimes-Duenez et al., 2015
KM203270.1	Haplotype 22	*Aedes aegypti*	Colombia	Jaimes-Duenez et al., 2015
KM203271.1	Haplotype 23	*Aedes aegypti*	Colombia	Jaimes-Duenez et al., 2015
KM203272.1	Haplotype 24	*Aedes aegypti*	Colombia	Jaimes-Duenez et al., 2015
KM203273.1	Haplotype 25	*Aedes aegypti*	Colombia	Jaimes-Duenez et al., 2015
KM203274.1	Haplotype 26	*Aedes aegypti*	Colombia	Jaimes-Duenez et al., 2015
KM203275.1	Haplotype 27	*Aedes aegypti*	Colombia	Jaimes-Duenez et al., 2015
KM203276.1	Haplotype 28	*Aedes aegypti*	Colombia	Jaimes-Duenez et al., 2015
KM203277.1	Haplotype 29	*Aedes aegypti*	Colombia	Jaimes-Duenez et al., 2015
KM203278.1	Haplotype 30	*Aedes aegypti*	Colombia	Jaimes-Duenez et al., 2015
KM203279.1	Haplotype 31	*Aedes aegypti*	Colombia	Jaimes-Duenez et al., 2015
KM203280.1	Haplotype 32	*Aedes aegypti*	Colombia	Jaimes-Duenez et al., 2015
KM203281.1	Haplotype 33	*Aedes aegypti*	Colombia	Jaimes-Duenez et al., 2015
KM203282.1	Haplotype 34	*Aedes aegypti*	Colombia	Jaimes-Duenez et al., 2015
KM203283.1	Haplotype 35	*Aedes aegypti*	Colombia	Jaimes-Duenez et al., 2015
KM203284.1	Haplotype 36	*Aedes aegypti*	Colombia	Jaimes-Duenez et al., 2015
KM203285.1	Haplotype 37	*Aedes aegypti*	Colombia	Jaimes-Duenez et al., 2015
KM203286.1	Haplotype 38	*Aedes aegypti*	Colombia	Jaimes-Duenez et al., 2015
KM203287.1	Haplotype 39	*Aedes aegypti*	Colombia	Jaimes-Duenez et al., 2015
KM203288.1	Haplotype 40	*Aedes aegypti*	Colombia	Jaimes-Duenez et al., 2015
KM203289.1	Haplotype 41	*Aedes aegypti*	Colombia	Jaimes-Duenez et al., 2015
KM203290.1	Haplotype 42	*Aedes aegypti*	Colombia	Jaimes-Duenez et al., 2015
KM203291.1	Haplotype 43	*Aedes aegypti*	Colombia	Jaimes-Duenez et al., 2015
KM203292.1	Haplotype 44	*Aedes aegypti*	Colombia	Jaimes-Duenez et al., 2015
KM203293.1	Haplotype 45	*Aedes aegypti*	Colombia	Jaimes-Duenez et al., 2015
KM203294.1	Haplotype 46	*Aedes aegypti*	Colombia	Jaimes-Duenez et al., 2015
KM203295.1	Haplotype 47	*Aedes aegypti*	Colombia	Jaimes-Duenez et al., 2015
KM203296.1	Haplotype 48	*Aedes aegypti*	Colombia	Jaimes-Duenez et al., 2015
KM203297.1	Haplotype 49	*Aedes aegypti*	Colombia	Jaimes-Duenez et al., 2015
KM203298.1	Haplotype 50	*Aedes aegypti*	Colombia	Jaimes-Duenez et al., 2015
KM203299.1	Haplotype 51	*Aedes aegypti*	Colombia	Jaimes-Duenez et al., 2015
KM203300.1	Haplotype 52	*Aedes aegypti*	Colombia	Jaimes-Duenez et al., 2015
KM203301.1	Haplotype 53	*Aedes aegypti*	Colombia	Jaimes-Duenez et al., 2015
KM203302.1	Haplotype 54	*Aedes aegypti*	Colombia	Jaimes-Duenez et al., 2015
KM203303.1	Haplotype 55	*Aedes aegypti*	Colombia	Jaimes-Duenez et al., 2015
KM203304.1	Haplotype 56	*Aedes aegypti*	Colombia	Jaimes-Duenez et al., 2015
KM203305.1	Haplotype 57	*Aedes aegypti*	Colombia	Jaimes-Duenez et al., 2015
KM203306.1	Haplotype 58	*Aedes aegypti*	Colombia	Jaimes-Duenez et al., 2015
KM203307.1	Haplotype 59	*Aedes aegypti*	Colombia	Jaimes-Duenez et al., 2015
KM203308.1	Haplotype 60	*Aedes aegypti*	Colombia	Jaimes-Duenez et al., 2015
KM203309.1	Haplotype 61	*Aedes aegypti*	Colombia	Jaimes-Duenez et al., 2015
KM203310.1	Haplotype 62	*Aedes aegypti*	Colombia	Jaimes-Duenez et al., 2015

KM203311.1	Haplotype 63	*Aedes aegypti*	Colombia	Jaimes-Duenez et al., 2015
KM203312.1	Haplotype 64	*Aedes aegypti*	Colombia	Jaimes-Duenez et al., 2015
KM203313.1	Haplotype 65	*Aedes aegypti*	Colombia	Jaimes-Duenez et al., 2015
KM203314.1	Haplotype 66	*Aedes aegypti*	Colombia	Jaimes-Duenez et al., 2015
KM203315.1	Haplotype 67	*Aedes aegypti*	Colombia	Jaimes-Duenez et al., 2015
KM203316.1	Haplotype 68	*Aedes aegypti*	Colombia	Jaimes-Duenez et al., 2015
KM203317.1	Haplotype 69	*Aedes aegypti*	Colombia	Jaimes-Duenez et al., 2015
KM203318.1	Haplotype 70	*Aedes aegypti*	Colombia	Jaimes-Duenez et al., 2015
KM203319.1	Haplotype 71	*Aedes aegypti*	Colombia	Jaimes-Duenez et al., 2015
KM203320.1	Haplotype 72	*Aedes aegypti*	Colombia	Jaimes-Duenez et al., 2015
KM203321.1	Haplotype 73	*Aedes aegypti*	Colombia	Jaimes-Duenez et al., 2015
KM203322.1	Haplotype 74	*Aedes aegypti*	Colombia	Jaimes-Duenez et al., 2015
KM203323.1	Haplotype 75	*Aedes aegypti*	Colombia	Jaimes-Duenez et al., 2015
KM203324.1	Haplotype 76	*Aedes aegypti*	Colombia	Jaimes-Duenez et al., 2015
KM203325.1	Haplotype 77	*Aedes aegypti*	Colombia	Jaimes-Duenez et al., 2015
KM203326.1	Haplotype 78	*Aedes aegypti*	Colombia	Jaimes-Duenez et al., 2015
KM203327.1	Haplotype 79	*Aedes aegypti*	Colombia	Jaimes-Duenez et al., 2015
KM203328.1	Haplotype 80	*Aedes aegypti*	Colombia	Jaimes-Duenez et al., 2015
KM203329.1	Haplotype 81	*Aedes aegypti*	Colombia	Jaimes-Duenez et al., 2015
KM203330.1	Haplotype 82	*Aedes aegypti*	Colombia	Jaimes-Duenez et al., 2015
KM203331.1	Haplotype 83	*Aedes aegypti*	Colombia	Jaimes-Duenez et al., 2015
KM203332.1	Haplotype 84	*Aedes aegypti*	Colombia	Jaimes-Duenez et al., 2015
KM203333.1	Haplotype 85	*Aedes aegypti*	Colombia	Jaimes-Duenez et al., 2015
KM203334.1	Haplotype 86	*Aedes aegypti*	Colombia	Jaimes-Duenez et al., 2015
KM203335.1	Haplotype 87	*Aedes aegypti*	Colombia	Jaimes-Duenez et al., 2015
KM203336.1	Haplotype 88	*Aedes aegypti*	Colombia	Jaimes-Duenez et al., 2015
EU650405.1	Haplotype 1	*Aedes aegypti*	Brazil	Lima and Scarpassa, 2009
EU650406.1	Haplotype 2	*Aedes aegypti*	Brazil	Lima and Scarpassa, 2009
EU650407.1	Haplotype 3	*Aedes aegypti*	Brazil	Lima and Scarpassa, 2009
EU650408.1	Haplotype 4	*Aedes aegypti*	Brazil	Lima and Scarpassa, 2009
EU650409.1	Haplotype 5	*Aedes aegypti*	Brazil	Lima and Scarpassa, 2009
EU650410.1	Haplotype 6	*Aedes aegypti*	Brazil	Lima and Scarpassa, 2009
EU650411.1	Haplotype 7	*Aedes aegypti*	Brazil	Lima and Scarpassa, 2009
EU650412.1	Haplotype 8	*Aedes aegypti*	Brazil	Lima and Scarpassa, 2009
EU650413.1	Haplotype 9	*Aedes aegypti*	Brazil	Lima and Scarpassa, 2009
EU650414.1	Haplotype 10	*Aedes aegypti*	Brazil	Lima and Scarpassa, 2009
EU650415.1	Haplotype 11	*Aedes aegypti*	Brazil	Lima and Scarpassa, 2009
EU650416.1	Haplotype 12	*Aedes aegypti*	Brazil	Lima and Scarpassa, 2009
EU650417.1	Haplotype 13	*Aedes aegypti*	Brazil	Lima and Scarpassa, 2009
AY906835.1	Clone 1	*Aedes aegypti*	Brazil	Paduan and Ribolla, 2008
AY906836.1	Clone 2	*Aedes aegypti*	Brazil	Paduan and Ribolla, 2008
AY906837.1	Clone 3	*Aedes aegypti*	Brazil	Paduan and Ribolla, 2008
AY906838.1	Clone 4	*Aedes aegypti*	Brazil	Paduan and Ribolla, 2008

AY906839.1	Clone 5	*Aedes aegypti*	Brazil	Paduan and Ribolla, 2008
AY906840.1	Clone 6	*Aedes aegypti*	Brazil	Paduan and Ribolla, 2008
AY906841.1	Clone 7	*Aedes aegypti*	Brazil	Paduan and Ribolla, 2008
AY906842.1	Clone 8	*Aedes aegypti*	Brazil	Paduan and Ribolla, 2008
AY906843.1	Clone 9	*Aedes aegypti*	Brazil	Paduan and Ribolla, 2008
AY906844.1	Clone 10	*Aedes aegypti*	Brazil	Paduan and Ribolla, 2008
AY906845.1	Clone 11	*Aedes aegypti*	Brazil	Paduan and Ribolla, 2008
AY906846.1	Clone 13	*Aedes aegypti*	Brazil	Paduan and Ribolla, 2008
AY906847.1	Clone 12	*Aedes aegypti*	Brazil	Paduan and Ribolla, 2008
AY906848.1	Clone 14	*Aedes aegypti*	Brazil	Paduan and Ribolla, 2008
AY906849.1	Clone 15	*Aedes aegypti*	Brazil	Paduan and Ribolla, 2008
AY906850.1	Clone 16	*Aedes aegypti*	Brazil	Paduan and Ribolla, 2008
AY906851.1	Clone 17	*Aedes aegypti*	Brazil	Paduan and Ribolla, 2008
AY906852.1	Clone 18	*Aedes aegypti*	Brazil	Paduan and Ribolla, 2008
AY906853.1	Clone 19	*Aedes aegypti*	Brazil	Paduan and Ribolla, 2008
JQ926705.1	Haplotype 1	*Aedes aegypti*	Bolivia	Paupy et al., 2012
JQ926706.1	Haplotype 2	*Aedes aegypti*	Bolivia	Paupy et al., 2012
JQ926707.1	Haplotype 3	*Aedes aegypti*	Bolivia	Paupy et al., 2012
JQ926708.1	Haplotype 4	*Aedes aegypti*	Bolivia	Paupy et al., 2012
MK359818.1	CV1	*Aedes aegypti*	Cape Verde	Salgueiro et al., 2019
MK359819.1	CV2	*Aedes aegypti*	Cape Verde	Salgueiro et al., 2019
MK359820.1	CV3	*Aedes aegypti*	Cape Verde	Salgueiro et al., 2019
MK359821.1	CV4	*Aedes aegypti*	Cape Verde	Salgueiro et al., 2019
MK359822.1	CV5	*Aedes aegypti*	Cape Verde	Salgueiro et al., 2019
MK359823.1	CV6	*Aedes aegypti*	Cape Verde	Salgueiro et al., 2019
MK359824.1	CV7	*Aedes aegypti*	Cape Verde	Salgueiro et al., 2019
JN089748.1	Isolate A	*Aedes aegypti*	Brazil - Parana	Twerdochlib et al., 2012
JN089749.1	Isolate B	*Aedes aegypti*	Brazil - Parana	Twerdochlib et al., 2012
JN089750.1	Isolate C	*Aedes aegypti*	Brazil - Parana	Twerdochlib et al., 2012
JN089751.1	Isolate D	*Aedes aegypti*	Brazil - Parana	Twerdochlib et al., 2012
JN089752.1	Isolate E	*Aedes aegypti*	Brazil - Parana	Twerdochlib et al., 2012
JN089753.1	Isolate F	*Aedes aegypti*	Brazil - Parana	Twerdochlib et al., 2012
JN089754.1	Isolate G	*Aedes aegypti*	Brazil - Parana	Twerdochlib et al., 2012
JN089755.1	Isolate H	*Aedes aegypti*	Brazil - Parana	Twerdochlib et al., 2012
